# Burnout and Satisfaction with Work–Life Balance among General Practitioners in Bulgaria during the COVID-19 Pandemic

**DOI:** 10.3390/healthcare12100987

**Published:** 2024-05-10

**Authors:** Kristina Kilova, Rumyana Stoyanova, Stanislava Harizanova, Marin Baltov

**Affiliations:** 1 Department of Medical Informatics, Biostatistics and E-Learning, Medical University of Plovdiv, 4002 Plovdiv, Bulgaria; kristina.kilova@mu-plovdiv.bg; 2 Department of Health Management and Health Economics, Medical University of Plovdiv, 4002 Plovdiv, Bulgaria; 3 Department of Hygiene, Medical University of Plovdiv, 4002 Plovdiv, Bulgaria; stanislava.harizanova@mu-plovdiv.bg; 4 Department of Forensic Medicine and Deontology, Medical University of Plovdiv, 4002 Plovdiv, Bulgaria; marin.baltov@mu-plovdiv.bg

**Keywords:** general practitioners, work–life balance, professional stress, professional life, coronavirus pandemics

## Abstract

The objective of the present study is to analyze the link between the degree of professional burnout among general practitioners in Bulgaria during a pandemic and their satisfaction with the balance between their personal lives and professional lives. A cross-sectional study was conducted during the fourth wave of the COVID-19 pandemic from December 2021 to January 2022 among 377 general practitioners. We identified the presence and level of burnout syndrome among GPs using V. Boyko’s method for diagnostics of the severity of symptoms and the phases of formation and completion of the ‘occupational burnout’ process. Descriptive statistics and parametric and non-parametric tests were used for the analysis. For the significance level of the null hypothesis, we assumed that *p* < 0.05 at a 95% confidence interval. A total of 96.3% of the GPs had a high level of burnout during the COVID-19 pandemic. They worked more than 8 h a day (74.8%) and more than 5 days a week (69.0%). A total of 86.3% of them were not satisfied with the spare time they had and stated that they could not balance their work and personal lives (67.1%) since the pandemic was announced. A link was found between the level of professional burnout and long working hours (*p* = 0.022), dissatisfaction with free time (*p* = 0.028), and the inability to balance work and personal life (*p* = 0.000), as well as concerns related to safety during the pandemic (*p* = 0.048). Unrealistically high levels of burnout during the COVID-19 pandemic due to a disturbed work–life balance is a reason to re-evaluate health policies and involve more hospital care doctors at the frontlines to fight against severe infectious diseases. The results of this study could be used to inform policy makers, healthcare managers, and other stakeholders about the factors that have had profound impacts on GPs’ stress levels during the COVID-19 pandemic.

## 1. Introduction

Achieving a satisfactory balance between time spent at work and leisure in the life of a modern person is a topic that has excited researchers, employers, trade unions, and politicians for many years. The main determinants that are generally tested when assessing enjoyment with these two variables are satisfaction with the current job, with payment, and with the amount of spare time available. In recent years, balancing personal life and professional life has become increasingly important, as time is regarded as an extremely valuable resource related to both work and leisure [1].

Many studies have proven that there is a link between the satisfaction of individuals and the lengths of their working days and the amount of spare time they have; this is the so-called balance between personal life and professional life. Some evidence suggests that long working hours jeopardize work–life balance [2,3]. On the other hand, reducing working time does not always improve this balance, because transforming it into more spare time is not a guarantee that it will be well spent [1,4]. Individuals’ satisfaction with leisure not only depends on its duration, but also on its quality, i.e., how beneficial it is for people and their loved ones. Other studies have proven that people who are pleased with their current jobs and payment, respectively, are significantly more satisfied with the spare time they have, as well as with its quality, regarding their personal preferences [5]. A harmonious balance between work and personal life is crucial for building a healthy and stress-free environment and to fully leverage employees’ potential [6,7]. The number of working hours can be considered as a factor that increases people’s well-being and stimulates the internal and external demands for goods and services, and it is a prerequisite for spare time to be well spent [8]. There is evidence that spare time, in turn, contributes to improving the health statuses and well-being of individuals as well as reducing stress in everyday life [9].

Stress is a serious issue in modern society, and it is closely related to both lifestyle and work duties. Stress at the workplace is reflected in an insufficient application of work skills and negatively affects the functioning of an organization as a whole. Some of the major health care stressors reported in the scientific literature are long work hours, frequently changing demands and responsibilities, weekend work, patient-care-related stress, and an overall unsafe work environment. Prolonged exposure to stress leads to professional burnout. Burnout syndrome is manifested in emotional exhaustion, frustration, indifference to patients, a reduced desire to work, a lower self-esteem, and even the act of leaving a profession. In a medical environment, it negatively affects the attention paid to patients, which lowers the quality of medical care [4,10,11]. Some studies in the medical field have analyzed the relationship between sociodemographic, professional, and personal variables and the occurrence of burnout syndrome [12,13].

General practitioners are at significant risk of stress, burnout, and mental health problems such as anxiety and depression. The peril is greater than that of the rest of the working population and increases over time. This is a consequence of the growing expectations of society and the faster paces of life and work.

Poor mental well-being and low work satisfaction among medical specialists can have major negative consequences for them and their patients and can also reduce the cost-effectiveness of health care. Mental distress is becoming increasingly common among physicians, including general practitioners (GPs) [14]. They are more vulnerable to burnout (especially emotional exhaustion), stress, and mental health problems than doctors of all other specialties [15]. This is related to the increased demands on primary care along with decreasing financial and personnel resources [16,17].

The COVID-19 pandemic restrictions have had a significant impact on the work of medical specialists, creating a number of challenges and increasing professional stress [18]. The pandemic has brought to light the actual deficiencies of national healthcare systems [19,20]. General practitioners are victims of the global phenomenon better known by the term ‘great resignation’, the leading cause of burnout [21], which involves feelings of exhaustion, alienation, cynicism, or pessimism toward one’s work, along with reduced job performance, resulting from chronic stress in the workplace [14,21,22].

The COVID-19 pandemic exacerbated existing problems by also creating new occupational stressors for GPs, namely an increased risk of exposure to SARS-CoV-2 [23]. General practitioners, who are already in a profession with a high workload, were at the frontlines of providing COVID-19-related healthcare in addition to routine care. In these extreme conditions, not only did they have to be on the frontlines in their offices, but they also had to be constantly available to their patients [24], which means they very often exceeded the limits of their fixed working hours [25]. One of the more frequently identified factors in physician burnout is working long hours. Most GPs perceived an increase in consultation frequency, consultation times, and workload since the outbreak. More vaccination consultations meant reduced home visits, acute consultation times, and cancer screenings. Pandemic-related bureaucracy, restricted access to therapy and rehabilitation services specialized for COVID-19, unreliable vaccine deliveries, mandatory telematics infrastructure implementation, and frequent changes in official regulations were the main reasons for GPs’ dissatisfaction [26]. The inability to balance work and personal life due to excessively high work demands also increases the risk of emotional exhaustion and distress among GPs [27,28]. A number of reports have described the physical and psychological morbidity among healthcare professionals during the COVID-19 pandemic [29,30,31]. One of the essential preventive measures to improve the mental health of general practitioners, especially in the context of the COVID-19 pandemic, is to increase their time for rest and relaxation between shifts [32]. By reducing time spent on clerical tasks, an overall work–life balance shift can be achieved, enabling physicians time to recuperate and engage in self-care between and after shifts.

Psychological interventions, like providing access to clinically trained therapists, surveys to analyze levels of stress and anxiety, and yoga and meditation workshops, were found to be relatively successful in burnout reduction [33]. The impact of these interventions could increase the mental health of general practitioners in the post-pandemic era. Burnout is a complicated problem and should be treated by combining interventions. It is necessary to build habits and opportunities to maintain the physical needs of general practitioners (short breaks and exercises), control overwork and excessive workloads, increase intercollegiate support, and raise awareness of the causes and risks of experiencing burnout [14].

This study is focused on the dependence between work–life balance and professional burnout among general practitioners in Bulgaria during the COVID-19 pandemic. This will make it possible to measure and evaluate burnout development in conditions of hard-to-manage cataclysms that have a serious impact on professional life and personal life. The results of such a study would not only be helpful for the scientific literature to prove or disprove the link between the course and management of pandemic events and the development of burnout, but they would also be useful for government authorities to take more adequate measures to manage such critical processes.

The objective of the present study is to analyze the link between the degree of professional burnout of general practitioners in Bulgaria during a pandemic and their satisfaction from the balance between personal life and professional life.

## 2. Materials and Methods

### 2.1. Procedure

The present study was conducted among general practitioners during the fourth wave of the COVID-19 pandemic, from December 2021 to January 2022, to examine the relationship between the degree of professional burnout and satisfaction from the balance between personal life and professional life.

The current work used a cross-sectional survey design in order to collect data from many subjects at a single point of time. The study participants were selected using a random sampling method. The National Health Insurance Fund contract partners list was used with random number assignment and selection using a stepwise approach. According to the National Statistical Institute of the Republic of Bulgaria (NSI), there were 4015 GPs in Bulgaria by 31 December 2020. The GP sample size was calculated at a maximum variance of 50% with a 95% confidence interval, and it was bounded to a maximum error of 5%. The sample size was set at 351 GPs. Based on the literature review, the average response rate to email surveys among physicians is 35% [34]. Thus, 1200 GPs were selected, representing 30% of all GPs in Bulgaria. The selected GPs received an invitation to fill out an online questionnaire, prepared with the aid of Google Forms^®^ 0.8, using their emails or Viber numbers provided by the website of the National Health Insurance Fund. Participation was voluntary, individual, and anonymous without any financial compensation. All requirements regarding confidentiality of medical and personal data were strictly followed when collecting and analyzing the data according to the General Data Protection Regulations (GDPR) (EU) 2016/679 issued by the European Parliament.

### 2.2. Sample

The number of validated responses was 377, which represented almost 10% of all the GPs in Bulgaria. The sample included 78.2% female participants and 21.8% males. The response rate was 31.4%. The number of respondents is sufficient to conclude that the results are statistically significant.

### 2.3. Measures

The principal tool used for data collection was the specially designed questionnaire organized in three sections. The first section covered demographic characteristics, such as age, gender, marital status, number of children in the family under 18, total work experience, work experience as GP, and type of practice.

The second section identified the presence and level of burnout syndrome among GPs using V. Boyko’s method for diagnostics of the severity of symptoms and the phases of formation and completion of the ‘occupational burnout’ process. It contained 84 questions, each with a different weight [35]. The questionnaire was adapted and validated for the Bulgarian population [36].

The following two questionnaires were mainly used to study burnout syndrome in Bulgaria: the Maslach Burnout Inventory (MBI) and Boyko’s Burnout Inventory (BBI). Despite the fact that the questionnaires are based on different theoretical models and have unalike scales, they essentially evaluate the same construct. As opposed to Christina Maslach, Viktor V. Boyko used the term ‘emotional burnout’ and identified three phases in the formation of burnout syndrome, each corresponding to the stages of stress, as indicated by Hans Selye: alarm, resistance, and exhaustion. At each phase, a specific set of emotional burnout symptoms is formed. In fact, the concepts of ‘emotional burnout’, as presented by Boyko, and ‘burnout’, as presented by Maslach, are essentially identical. The comparison (MBI vs. BBI) demonstrated the consistency of the results, which implied the possibility of comparing data yielded by the studies based on the two questionnaires. The clarity and homogeneity of interpretation of the scales are significant advantages of Boyko’s method. The score obtained from this test can be easily compared to the results obtained using other psychological diagnostic techniques. All of these benefits make it an appropriate tool for assessing burnout in our study. Boyko’s Burnout Inventory (BBI) includes 84 items (statements), which form 12 scales (4 for each burnout phase). As a result of processing the answers on the basis of the scale scores, the dominant symptoms are identified, and the degree of formation of each of the three phases is determined. Items of the questionnaire scales have different weights associated with the contribution of a particular manifestation of the syndrome to its formation, and these differences were considered when calculating the final scores on each scale.

The internal consistency of all scales exceeds the standard of 0.70 (0.797–0.930), i.e., it is distinguished by very good psychometric indicators, which makes it reliable and applicable in the study of burnout syndrome among workers. In addition, an external validation of the proposed methodology was carried out by evaluating the link with the other well-known questionnaire that was validated and standardized for Bulgaria—Maslach’s Burnout Inventory (MBI). The results show that there was a moderate to strong correlation between the most commonly used questionnaire for the assessment of burnout and Boyko’s method, which we propose here [35].

The third part of the questionnaire covered eleven closed questions and one open-ended question to ascertain the length of the working week. A 5-point Likert scale was applied to assess the respondents’ levels of satisfaction with the ‘work/spare time’ ratio since the announcement of the COVID-19 pandemic.

### 2.4. Data Analysis

Statistical data processing was performed using the software product SPSS v.22.0. Descriptive statistics was used to review the frequency, mean, and standard deviation (SD) for the demographic characteristics. To study the continuous variables that had normal distribution, Student’s *t*-test and an ANOVA test were used. Continuous variables with a non-normal distribution were compared with the Kruskal–Wallis test and the Mann–Whitney U test. The link between categorical variables was analyzed using a χ^2^ test. Correlation analysis was performed by employing either Pearson’s correlation coefficient or Spearman’s rho according to the normality of continuous variables. Central tendencies were presented with mean (M) and standard deviation (SD). For the significance level of the null hypothesis, we assumed that *p* < 0.05 at a 95% confidence interval.

## 3. Results

Table 1 presents the demographic characteristics of the GPs. As can be seen from the data, women (78.2%) (*n* = 295) and married people (71.4%) (*n* = 269) predominated the sample. Almost ¾ of the respondents did not have children under the age of 18—specifically 74.0% (*n* = 279). A significant share of them had an individual practice—specifically 82.8% (*n* = 312). They had extensive professional experience—on average, 29.30 ± 6.980 years—and their length of experience as general practitioners was 19.10 ± 4.354 years. The average duration of their working week was 48.98 ± 16.054 h.

The results show that 96.3% (n = 363) of the GPs had a high level of burnout during the fourth wave of the COVID-19 pandemic. Table 2 shows the distribution of the respondents’ answers regarding the factors influencing work–life balance and the level of burnout.

During the pandemic, an essential number of GPs declared that they had to work more than 8 h a day (74.8%; *n* = 282) and more than 5 days a week (69.0%; *n* = 260), as well as during their inter-shift breaks (91.8%; *n* = 346) or leaves (71.6%; *n* = 270). A significant number of the respondents were not satisfied with the spare time they had (86.3%; *n* = 325) and believed they could not find a balance between work and personal life (67.1%; *n* = 244) since the pandemic had been announced. A non-parametric analysis revealed that there was a link between the level of burnout and long working hours (*p* = 0.022), dissatisfaction with leisure time (*p* = 0.028), and the inability to balance work and personal life (*p* = 0.000), as well as safety concerns during the pandemic (*p* = 0.048).

It was ascertained that there was a statistical dependence in the levels of burnout syndrome by gender (*p* = 0.009). No differences were found concerning professional experience, marital status, the number of children under 18, and type of practice.

The general practitioners who were pleased with the payment they received during the pandemic were also satisfied with their current workplaces (χ^2^ = 46.860; *p* = 0.000; r_s_ = 0.536), confirming the major link between work satisfaction and payment. Too much leisure time (more than 8 h) as well as insufficient leisure time (less than 4 h) led to an increase in the respondents’ dissatisfaction with the studied variable (χ^2^ = 53.891; *p* = 0.034; r_s_ = −0.281).

## 4. Discussion

This study discovered that there was a great proportion of individuals with a high level of burnout during the fourth wave of the COVID-19 pandemic. A meta-analysis of the prevalence of burnout among GPs, including results from 31 studies in the pre-pandemic period, revealed a high level of burnout on average among about 6% of the respondents and moderate general burnout in 32% [37].

A study focusing on burnout and the psychological outcomes of the COVID-19 outbreak in different professions of frontline HCWs and predictive factors of burnout also identified a low to moderate level of burnout at the beginning of the COVID-19 pandemic. However, they suggested that in the later stages of the pandemic, the burnout level of HCWs might be further adversely affected by increases in the number of cases and death rates, which is associated with greater workloads and more intensive work [38]. In this regard, our results prove that the changed working conditions in the midst of the pandemic had a negative impact on the development of burnout among the study’s participants.

Many studies confirmed that work–life balance was positively related to well-being, as well as work and life satisfaction, and it was negatively related to anxiety, depression, and mental health problems [39,40,41].

Our results were consistent with those of a study by Bodendieck et al. [42] that was conducted among GPs in Germany, namely that work–life balance was related to all dimensions of burnout, which partly determines GPs’ motivation to stay in the profession. Our results were also coherent with other studies showing associations between overtime, long shifts, and burnout among medical professionals [43,44].

Some studies proved that work satisfaction depends, to a significant extent, on the length of the working day, which, in turn, depends on the amount of spare time [23,30]. Authors of another study revealed an insignificant correlation between work satisfaction and leisure time, which does not contradict our results, where a similar dependence was also established [11].

Based on socio-economic data from the German Socio-Economic Panel Study over a 15-year period, with the help of mathematical modeling, researchers found that one standard additional hour of work in an actual working day led to a decrease in the satisfaction of individuals regarding their quality of work and life because it reduced the time they could spend with their families or for the benefit of themselves [45]. According to a survey conducted among 15 European countries, the average spare time available to individuals was between 4 and 6 h per day, which was comparable and did not contradict the results of our survey [46]. General practitioners are, at large, short on personal time, but during the COVID-19 pandemic, their personal time was even more limited. They had to work more than 8 h a day. They stated that they did not devote enough time to their families, and their balance between work and personal spare time was disturbed. Rodriguez et al. [47] reported that, during the COVID-19 pandemic, 90.8% of medical specialists changed their behaviors toward their families and friends, especially by reducing signs of attachment (76.8%).

As per an older study, women were more often dissatisfied with their work–life balance, which affected their emotional exhaustion. Such findings were also confirmed in the present study [48].

A survey among 228 general practitioners in Hungary regarding the most challenging aspects of the pandemic showed that, for the majority of them, as in our case, these were related to an increased workload [32]. The authors linked regular recreation to lower levels of stress, along with higher levels of mental and physical well-being. They considered that during the stressful period of the pandemic, apart from psychosocial support and better adaptation to the situation, leisure time was the second most important resource [32].

An online survey conducted in Germany was sent to 1444 GPs who had been selected based on their availability via email. Even though two reminders were sent to non-responders after the initial email, only 143 German GPs in total participated in the survey (response rate—9.9%) [26]. In contrast, we received 377 responses to our survey out of 1200 sent emails (response rate—31.8%), which is very satisfactory. It is also worth mentioning that we only received data for 2 months (December 2021–January 2022), while the authors of the German study received data for 3 months (May 2021–July 2021).

### Limitation

This study has some limitations. First, because the survey respondents voluntarily completed the survey, only those who had available time during the pandemic responded. Although the study was conducted over a short period of time, namely during the COVID-19 pandemic, the response rate was satisfactory compared to other online studies.

The results presented in this paper are a good starting point for in-depth research in the future. It is worth continuing with longitudinal studies and introducing individual variables, such as personality traits and coping mechanisms. These can be regarded as moderators in the relationship between socio-demographic variables.

## 5. Conclusions

A disturbed balance between personal life and professional life during the COVID-19 pandemic among general practitioners, expressed in reduced rest time and maximally extended working time, led to a high risk of professional burnout. Introducing measures that improve the balance between personal leisure and professional life can be a way to not only improve the mental health of general practitioners, but also to increase the quality of patient care.

Unrealistically high levels of burnout during the COVID-19 pandemic due to disturbed work–life balance is a reason to re-evaluate health policies. As a result, GPs’ burden during pandemic situations can be reduced. Burnout is a complicated problem and should be treated by combining interventions. It is necessary to build habits and opportunities to maintain the physical needs of general practitioners (short breaks and exercises), to control overwork and excessive workloads, etc.

The results of this study could be used to inform policy makers, healthcare managers, and other stakeholders about the factors that have had profound impacts on GPs’ stress levels during the COVID-19 pandemic.

## Figures and Tables

**Table 1:** Demographic characteristics of respondents (N = 377).

Age	*n* (%)
under 35 years old	5 (1.3)
36–50 years old	88 (23.3)
51–63 years old	255 (67.6)
over 64 years old	29 (7.7)
**Mean (SD)**	**54.8 (6.6)**
**Gender**	
men	82 (21.8)
women	295 (78.2)
**Marital status**	
married	269 (71.4)
cohabitation	35 (9.3)
divorced	41 (10.9)
widower	17 (4.5)
unmarried	15 (4.0)
**Number of children under 18**	
0	279 (74.0)
1	66 (17.5)
2	30 (8.0)
3	1 (0.3)
4	1 (0.3)
**Type of practice**	
individual	312 (82.8)
group	65 (17.2)
**Professional experience**	
under 5 years	3 (0.8)
6–10 years	4 (1.1)
11–20 years	22 (5.8)
over 21 years	348 (92.3)
**Mean (SD)**	**29.3 (7.0)**
**Professional experience as GP**	
under 5 years	9 (2.4)
6–10 years	10 (2.7)
11–20 years	327 (86.7)
over 21 years	30 (8.0)
**Mean (SD)**	**19.1 (4.36)**
**Average weekly working hours**	
under 35 h	42 (11.1)
36–40 h	90 (23.9)
41–45 h	19 (5.0)
over 46 h	226 (59.9)
**Mean (SD)**	**49 (16.1)**

**Table 2:** The distribution of the GPs’ responses regarding the relationship between the factors influencing work–life balance and the level of burnout.

Questions	Answers	Level of Professional Burnout			*p*
		Low *n* (%)	Mean *n* (%)	High *n* (%)	
Have you had to work more than 5 days a week since the COVID-19 pandemic?	Never	0 (0.0)	0 (0.0)	16 (4.2)	*p* > 0.05
	Rarely	0 (0.0)	2 (0.5)	27 (7.2)	
	Sometimes	2 (0.5)	2 (0.5)	60 (15.9)	
	Often	1 (0.3)	5 (1.3)	127 (33.7)	
	Always	0 (0.0)	2 (0.5)	133 (35.3)	
Have you had to work more than 8 h a day since the outbreak of the COVID-19 pandemic?	Never	0 (0.0)	1 (0.3)	15 (4.0)	*p* < 0.05 *
	Rarely	0 (0.0)	0 (0.0)	18 (4.8)	
	Sometimes	3 (0.8)	3 (0.8)	48 (12.7)	
	Often	0 (0.0)	5 (1.3)	149 (39.5)	
	Always	0 (0.0)	2 (0.5)	133 (35.3)	
How often have you found yourself working from home before or after normal working hours since the COVID-19 pandemic was announced?	Never	0 (0.0)	0 (0.0)	1 (0.3)	*p* > 0.05
	Rarely	0 (0.0)	0 (0.0)	4 (1.1)	
	Sometimes	0 (0.0)	2 (0.5)	12 (3.2)	
	Often	1 (0.3)	1 (0.3)	53 (14.1)	
	Always	2 (0.5)	8 (2.1)	293 (77.7)	
How often do you find yourself working on leave/vacation?	Never	0 (0.0)	2 (0.5)	17 (4.5)	*p* > 0.05
	Rarely	1 (0.3)	2 (0.5)	61 (16.2)	
	Sometimes	1 (0.3)	0 (0.0)	15 (4.0)	
	Often	1 (0.3)	1 (0.3)	75 (19.9)	
	Always	0 (0.0)	6 (1.6)	195 (51.7)	
Do you work shifts?	Never	1 (0.3)	3 (0.8)	93 (24.7)	*p* > 0.05
	Rarely	0 (0.0)	1 (0.3)	19 (5.0)	
	Sometimes	0 (0.0)	2 (0.5)	21 (5.6)	
	Often	0 (0.0)	0 (0.0)	27 (7.2)	
	Always	2 (0.5)	5 (1.3)	203 (53.8)	
I work flexible hours	Strongly disagree	0 (0.0)	1 (0.3)	19 (5.0)	*p* > 0.05
	Disagree	0 (0.0)	1 (0.3)	64 (17.0)	
	Neither agree nor disagree	1 (0.3)	6 (1.6)	156 (41.4)	
	Agree	1 (0.3)	2 (0.5)	71 (18.5)	
	Strongly agree	1 (0.3)	1 (0.3)	53 (14.1)	
I have additional leave	Strongly disagree	3 (0.8)	10 (2.7)	281 (74.5)	*p* > 0.05
	Disagree	0 (0.0)	0 (0.0)	33 (8.8)	
	Neither agree nor disagree	0 (0.0)	1 (0.3)	10 (2.7)	
	Agree	0 (0.0)	0 (0.0)	23 (6.1)	
	Strongly agree	0 (0.0)	0 (0.0)	16 (4.2)	
Do you feel that you haven’t been spending enough time with your family since the COVID-19 pandemic was announced?	Never	1(0.3)	1 (0.3)	9 (2.4)	*p* < 0.05 *
	Rarely	1 (0.3)	3 (0.8)	31 (8.2)	
	Sometimes	1 (0.3)	3 (0.8)	58 (14.9)	
	Often	0 (0.0)	2 (0.5)	176 (46.7)	
	Always	0 (0.0)	2 (0.5)	91 (24.1)	
How often do you think or worry about your safety at work?	Never	1 (0.3)	0 (0.0)	4 (1.1)	*p* < 0.05 *
	Rarely	0 (0.0)	1 (0.3)	21 (5.6)	
	Sometimes	0 (0.0)	1 (0.3)	31 (8.2)	
	Often	1 (0.3)	7 (1.9)	112 (29.7)	
	Always	1 (0.3)	2 (0.5)	195 (51.7)	
Do you feel like you cannot balance between your work and personal life since the COVID-19 pandemic was announced?	Never	0 (0.0)	1 (0.3)	5 (1.3)	*p* < 0.05 *
	Rarely	2 (0.5)	5 (1.3)	27 (7.2)	
	Sometimes	0 (0.0)	3 (0.8)	78 (20.7)	
	Often	0 (0.0)	1 (0.3)	170 (47.5)	
	Always	1 (0.3)	1 (0.3)	74 (19.6)	
Are you satisfied with the personal spare time you have available since the COVID-19 pandemic has been announced?	No	1 (0.3)	5 (1.3)	220 (58.4)	*p* < 0.05 *
	Rather no	0 (0.0)	4 (1.1)	105 (27.9)	
	I cannot decide	0 (0.0)	0 (0.0)	14 (3.7)	
	Rather yes	1 (0.3)	2 (0.5)	20 (5.3)	
	Yes	1 (0.3)	0 (0.0)	4 (1.3)	

* *p* < 0.05.

## Data Availability

Data are contained within the article.
